# Pancreatic cancer presenting with pulmonary cannonball lesions

**DOI:** 10.1016/j.radcr.2021.02.057

**Published:** 2021-03-17

**Authors:** Balraj Singh, Sydney Fasulo, Parminder Kaur, Beenish Faheen, Sarah Ayad, Sachin Gupta, Michael Maroules

**Affiliations:** a Hematology Oncology, Saint Joseph's University Medical Center, 703 Main street, Paterson, NJ 07503, USA; b Internal Medicine, Department of Internal Medicine, Tower Health Reading Hospital, West Reading, PA, USA

**Keywords:** Pancreatic cancer, Cannonball lesions, Malignancy, Lung metastasis, Cannonball metastases

## Abstract

Cannonball lesions are numerous, well-circumscribed, round pulmonary lesions and may be identified on plain radiograph or advanced imaging. This morphology can be associated with infectious causes, rheumatologic disease and metastatic disease. Classically, when cannonball lesions are associated with metastatic disease, they are seen in renal cell carcinoma and choriocarcinoma. We report a case of a 62-year-old Middle Eastern male who presented with shortness of breath, chest pain and fatigue and was found to have bilateral multiple pulmonary cannonball lesions and mass in the pancreas. Biopsy of one the lung lesions was consistent with pancreatic cancer. Our case adds to the limited literature available regarding cannonball lung lesions in the setting of pancreatic cancer. Health care providers should be aware of the various etiologies of cannonball lesions.

## Introduction

Pancreatic cancer is a major cause of cancer morbidity and mortality. It is considered to be one of the most aggressive and lethal neoplasms worldwide with overall 5-year survival of approximately 5% [1]. According to the International Agency for Research on Cancer (IARC) GLOBOCAN project, it is estimated that the incidence of pancreatic cancer in 2020 was 495,773 and the number of deaths in 2020 was 466,003 globally. In the United States alone, there were more than 62,643 new cases in 2020 and 53,277 deaths in 2020.

While the main causes of pancreatic cancer are not fully known, several risk factors have been reported. The most common risk factor is cigarette smoking, which accounts for the cause of 20%-25% of all pancreatic tumors. Other risk factors include advanced age, hereditary chronic pancreatitis, alcohol consumption, obesity, diabetes mellitus, and physical inactivity [1], [2], [3]. Typical presenting symptoms of pancreatic cancer include epigastric pain that radiates to the back, mid-back pain, jaundice, and weight loss [1,2]. Surgical resection is one of the treatment modalities; however, many pancreatic neoplasms are already at an advanced stage at detection and the anatomy of the tumor makes many unresectable resulting in an overall poor prognosis [1]. Most patients die with metastases to the liver, lung, and peritoneum [3]. Pancreatic metastatic disease of the lungs often presents as multiple pulmonary lesions; however, to our knowledge cannonball metastases have not been reported. A search of the PubMed did not reveal any case of pancreatic cancer presenting with cannonball lesions. Herein, we report a case of pulmonary cannonball metastases from pancreatic cancer.

## Case report

**Fig. 1:**
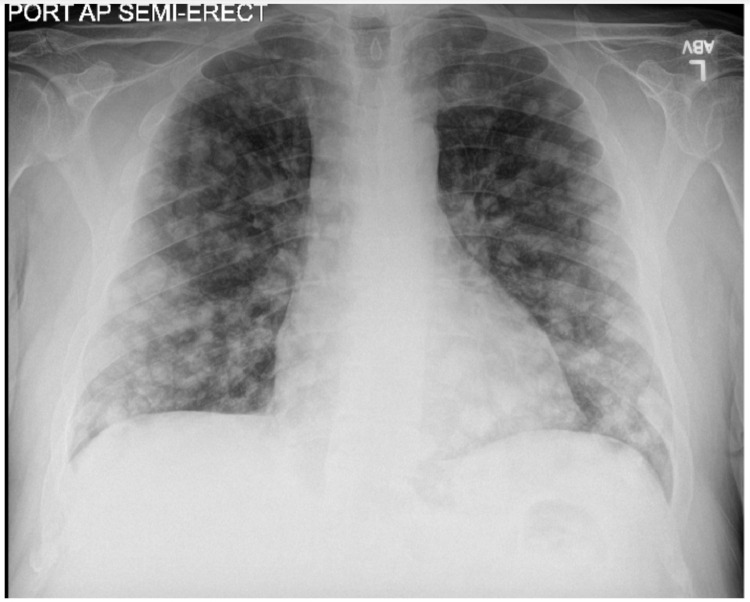
AP chest x-ray demonstrating extensive multifocal, round, nodular opacities compatible with metastatic disease.

**Fig. 2:**
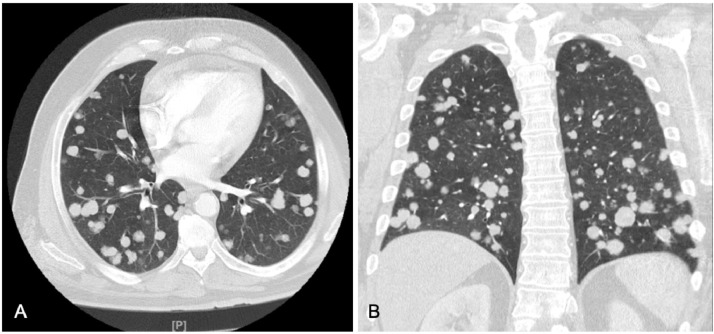
Axial (A) and coronal (B) lung window images from a CT with contrast demonstrating multiple bilateral round pulmonary metastasis.

**Fig. 3:**
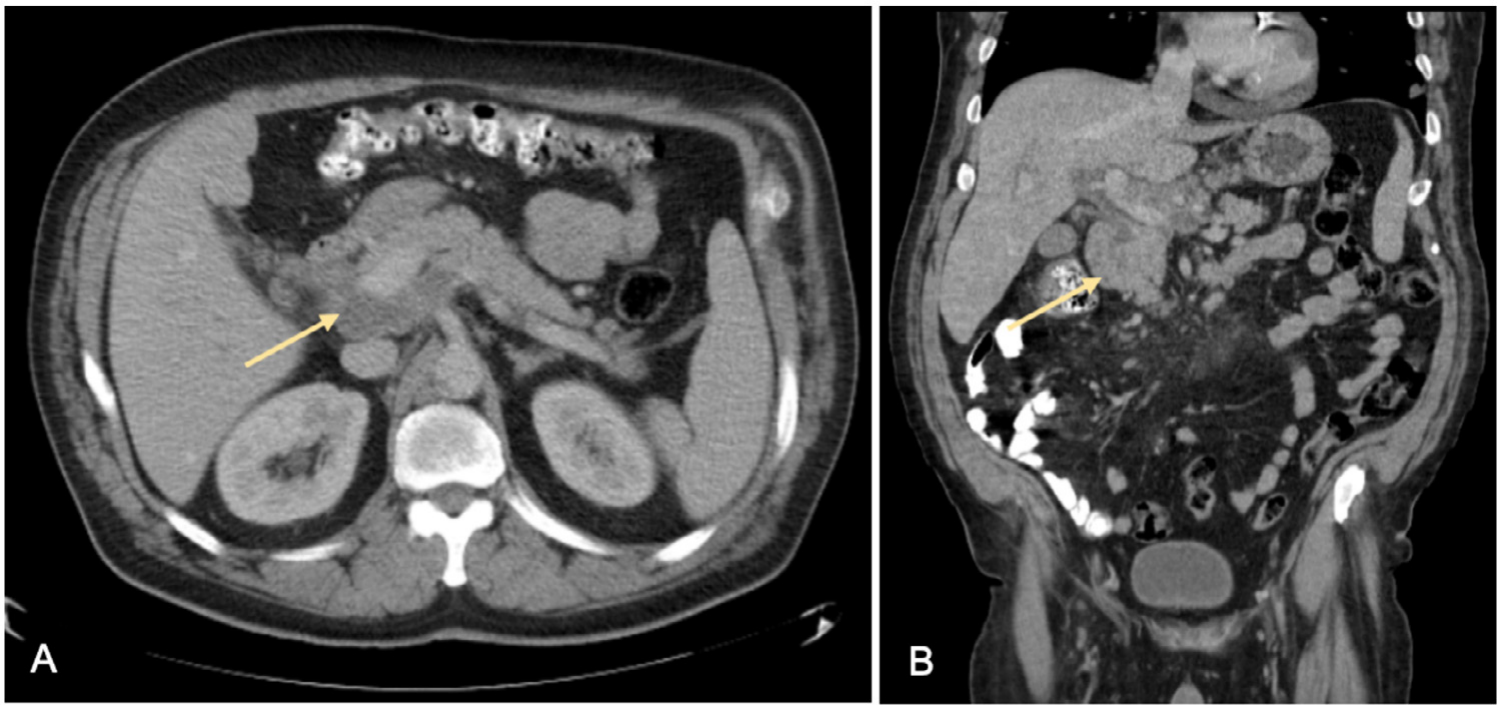
Axial (A) and coronal (B) images from a CT chest abdomen and pelvis with contrast showing a hypodense mass by the uncinate process of the pancreas measuring 5.6 by 2.7 cm.

We report a 62-year-old Middle Eastern male with a past medical history significant for hypertension, dyslipidemia, and smoking, who presented to the emergency department (ED) with complaints of progressively worsening shortness of breath on exertion, chest pain and fatigue of one-month duration. Vitals on presentation- temperature of 36.2°C, heart rate of 103 beats/minute, blood pressure of 119/75 mm Hg, respiratory rate of 18/min and saturating 100% on room air. Physical exam was unremarkable. The initial complete blood count demonstrated a white blood cell count of 10.1 × 10^9^/L (normal range: 4.5-11.0 × 10^9^/L), hemoglobin 7.2 g/dL (normal range: 13.5-17.5 g/dL), hematocrit 27.2% (normal range 41.0%-53.0%), platelets 352,000 mcL (normal range: 140,000-440,000 mcL). Brain natriuretic peptide and troponins were within normal limits. A chest x-ray revealed multiple, bilateral pulmonary nodules of various sizes (Fig. 1). A subsequent chest computed tomography (CT) with contrast showed multiple bilateral, large, well-circumscribed, round, pulmonary nodules also known as “cannonball” nodules (Fig. 2). This was suspicious for an underlying malignancy with metastasis. A full malignancy workup was undertaken and included a CT of the abdomen and pelvis with contrast which revealed a hypodense mass near the uncinate process of the pancreas measuring 5.6 cm by 2.7 cm and enlarged lymph nodes in the upper retroperitoneum measuring up to 3.2 cm x 2.4 cm (Fig. 3). The liver, spleen, and right adrenal gland were unremarkable.

The patient subsequently underwent biopsy of one of the cannonball lesions. This biopsy showed focal proliferation of mucinous glands and focal mucin extravasation. The mucinous glandular cells were cytologically bland in appearance; however, on immunostaining, the mucinous epithelial cells were positive for CK7, CK20, and CDX2; and negative for TTF-1 and Napsin A. The biopsy confirmed metastatic pancreatic adenocarcinoma. The patient was diagnosed with Stage IV pancreatic adenocarcinoma with lung metastasis. The patient was discharged to follow as an outpatient to start chemotherapy (modified chemotherapy regimen of leucovorin, fluorouracil, irinotecan, and oxaliplatin - FOLFIRINOX). Our case adds to the limited literature regarding pancreatic cancer presenting with cannonball metastasis to the lungs.

## Discussion

Classically, the term cannonball lesions are used for lung metastasis from a wide variety of causes, however the term has also been used to report skin metastasis in the setting of lung cancer and acquired tufted angioma due to their macroscopic appearance [4]. Pulmonary cannonball lesions have a distinctive radiographic appearance and are characterized by multiple solid, well-circumscribed, parenchymal masses of variable sizes [5]. When associated with metastatic disease, these pulmonary findings are a result of the hematogenous spread of disease to the lungs and representing an advanced stage and is associated with a poor overall survival.

Pulmonary cannonball metastases are usually seen in renal cell carcinoma, breast carcinoma, choriocarcinoma, endometrial cancer, prostate cancer, and head and neck squamous cell carcinoma [5,6]. There are a handful of case reports of other cancers that may present with metastatic cannonball lesions to the lungs which include melanoma, adrenocortical carcinoma, transitional cell carcinoma of the bladder, testicular cancer, and rarely gastric cancer [7,8]. In addition, hepatocellular carcinoma (HCC) rarely can metastasize to the right atrium via inferior vena cava. In such cases, HCC has extremely poor prognosis with fatal complications such as myocardial infarction, pulmonary embolism, and pulmonary cannonball metastases [9].

The patient might present with symptom's related to the metastatic cannonball lesions or the underlying primary tumor site depending upon the cause. In our case, the patient presented with shortness of breath and chest pain. Cannonball lesions may also be present in non-neoplastic conditions including fungal infections such as coccidioidomycosis and histoplasmosis, sarcoidosis, granulomatosis with polyangiitis (formerly Wegener's granulomatosis), nocardiosis, hydatid diseases, parasitic infections, rheumatoid disease, and pulmonary tuberculosis [5]. There have also been several reported cases of hemangiomas developing within the lungs, referred to as pulmonary cavernous hemangiomas giving the cannonball-like appearance, and can be misdiagnosed as lung metastases [10]. Sharma et al report a 40-year-old male with past medical history of rheumatic heart disease with severe mitral stenosis and Pulmonary hypertension who presented with fever, runny nose, sore throat, cough, exertional shortness of breath. Chest X ray showed three cannon ball shaped lesions in the right lung. Further imaging with chest computed tomography with contrast showed massively dilated pulmonary veins on right side giving the appearance of cannon ball lesions on chest x ray [11]. It is important to be aware of the various etiologies of cannonball lesions. Treatment is usually directed towards the underlying cause and symptom control.

## Conclusion

Cannonball lesions are seen in several disorders including rheumatological conditions, and various infections, however, they are most commonly associated with metastatic disease. Herein, we report a case of a 62-year-old male who was diagnosed with pancreatic cancer who presented with cannonball lesions. Our case report highlights the need for rigorous diagnostic workup for patients who present with this radiographic appearance.

## Patient Consent

Patient consent has been obtained
